# Impact of male-sterilizing doses and mating status on blood feeding rates and longevity in *Aedes aegypti*, *Aedes albopictus*, and *Anopheles arabiensis* females

**DOI:** 10.1186/s13071-025-07242-8

**Published:** 2026-03-21

**Authors:** Hanano Yamada, Nanwintoum Sévérin Bimbilé Somda, Béwadéyir Serge Poda, Carina Kraupa, Thomas Wallner, Wadaka Mamai, Kostas Bourtzis, Thabo Mashatola, Simran Singh Kotla, Chantel Janet de Beer, Fabrizio Balestrino, Jeremy Bouyer

**Affiliations:** 1 Insect Pest Control Section, Joint FAO/IAEA Centre of Nuclear Techniques in Food and Agriculture, Department of Nuclear Sciences and Applications, International Atomic Energy Agency, P.O. Box 100, 1400 Vienna, Austria; 2 Unité de Formation et de Recherche en Sciences et Technologies (UFR/ST), Université Norbert ZONGO (UNZ), BP 376, Koudougou, Burkina Faso; 3 Département de Biologie Médicale/Santé Publique (BioMed/SP), Institut de Recherche en Sciences de La Santé (IRSS), BP 545, Bobo-Dioulasso, Burkina Faso; 4 Experimental Zoology Group (EZO), Wageningen University & Research (WUR), 6708 WD Wageningen, The Netherlands; 5 Vector Reference Laboratory, Centre for Emerging Zoonotic and Parasitic Diseases, National Institute for Communicable Diseases, National Health, Laboratory Services, Johannesburg, South Africa; 6 ASTRE, CIRAD, INRAE, University of Montpellier, 34398 Montpellier, France; 7 ASTRE, CIRAD, INRAE, University of Montpellier, Plateforme Technologique CYROI, Sainte Clotilde, La Réunion, France

**Keywords:** Sterile insect technique, Vector competence, Irradiation, Blood feeding success, Sex separation

## Abstract

The irradiation of female mosquitoes has been shown to influence blood feeding behavior, depending on dose, life stage at irradiation, and mating status. In sterile male releases during the sterile insect technique (SIT) programs, a small number of sterile females can be released accidentally, which may pose a manageable but nonnegligible risk regarding disease transmission. In this study, female *Aedes aegypti, Aedes albopictus*, and *Anopheles arabiensis* were exposed to gamma irradiation at male-sterilizing doses and the blood feeding rates and longevity of mated versus unmated sterile females were assessed. Although some differences were observed between the three species, generally females irradiated at the pupal stage showed a marked decrease in blood feeding rates, while irradiated adults showed slightly higher feeding rates, more pronounced in the second week (for *Aedes* spp.). Overall, blood feeding females irradiated as pupae that were mated died soonest, while in the non-blood fed groups, irradiation only correlated with negative effects on survival in *Ae. aegypti*, and mating status correlated with diminished survival in *Ae. albopictus*. Although the overall risk of a small number of mated, irradiated females participating in disease transmission in SIT programs is expected to be very low, rigorous sex separation and quality control will minimize this risk, and developing genetic sexing strains to ensure safety should be prioritized.

**Graphical Abstract:**

**Figure:**
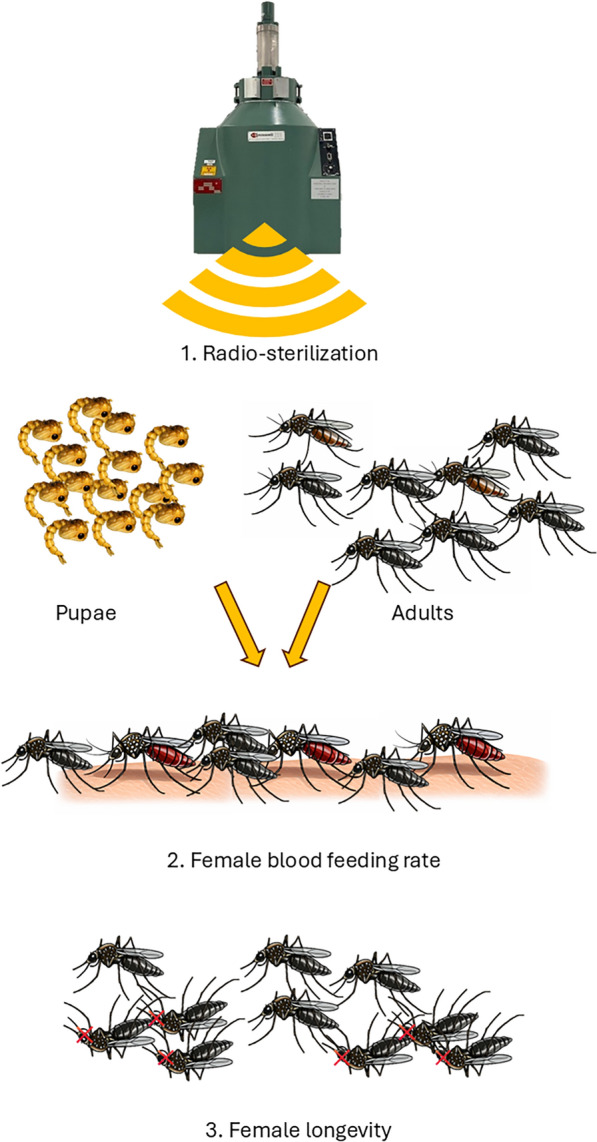

**Supplementary Information:**

The online version contains supplementary material available at 10.1186/s13071-025-07242-8.

## Background

The sterile insect technique (SIT) [1] is a species-specific insect pest control method based on the mass production and periodic release of sexually sterile insects. These sterile insects then seek and mate with wild counterparts and introduce sterility into the target population, thereby reducing the wild population with each generation. The technique has been implemented since the 1950s [2] and has celebrated many successes against numerous plant pests and human and animal disease vectors. It is generally advantageous to eliminate females before releasing sterile males, as operational costs can be reduced significantly, efficiency is enhanced as the sterile males will only be searching for wild females, and it is usually the females that cause damage to fruits or are potential disease vectors. The elimination of females prior to sterile male releases is an imperative component of SIT. This can be accomplished by visual, manual, mechanical, semi-automatic, and artificial intelligence (AI)-based sorting of either pupae or adults ([3–7], https://www.senecio-robotics.com/sit-hub), or by developing genetic sexing strains or genetically modified organisms (GMOs) [8–17]. The Insect Pest Control Laboratory of the Joint FAO/IAEA Programme of Nuclear Techniques in Food and Agriculture has been developing the SIT package against *Aedes aegypti, Aedes albopictus*, *Anopheles arabiensis*, [18] and more recently, *Anopheles stephensi*, important vectors of many arboviruses and malaria parasites. Although several genetic sexing strains are being developed for these species, improvements are always desirable, as no sexing system can guarantee the 100% elimination of females under large-scale rearing (or operational) conditions. This means that there is a risk of some females escaping the elimination process and being released together with the sterile males. In practice, many SIT programmes tolerate a female contamination rate of less than 1% during sterile male releases [19] on the basis of what is currently operationally achievable. These females will have been irradiated together with the males at doses required to achieve > 99% sterility in males. As females generally are significantly more radiosensitive, these doses incur enough damage to the female mosquitoes that they cease to lay eggs and will not contribute to the target population [20–23]. However, what impact does the irradiation exposure have on female blood feeding success that could affect pathogen transmission?

Irradiation of female *Aedes* mosquitoes has been shown to impact blood-feeding frequency differently on the basis of the species and the radiation dose [20, 22, 24]. Indeed, a study has shown that female *Ae. aegypti* irradiated at 30 Gy as mature pupae were completely sterilized, with reduced blood-feeding incidence at 50 Gy [25]. In addition, irradiation at doses ≥ 30 Gy, sufficient for male sterilization, significantly reduced female longevity, blood feeding, oviposition, and egg hatch rate [22]. A similar trend was observed in *An. arabiensis* females irradiated as pupae, which showed a significant reduction in blood feeding rates compared with unirradiated control females [21]. In contrast, Moretti et al. [20] found that, in *Ae. albopictus*, irradiation of pupae at 28 Gy led to a significant increase in the number of weekly bites per female compared with untreated controls, while doses of 35 and 45 Gy resulted in a tripling of the number of bites per female per week. The increase in the number of bites was confirmed in *Ae. aegypti* after irradiation at 50 Gy in the same study. The differences in feeding behavior observations can be attributed to methodological variations in assessing feeding propensity and the genetic backgrounds and length of colonization of the mosquito strains used in the studies. Moreover, irradiation can influence the transmission efficiency of arboviruses such as dengue and chikungunya, with varying effects on different *Aedes* species [26, 27]. While irradiation did not impact the transmission efficiency of these viruses, it did increase the virus load in both *Ae. albopictus* and *Ae. aegypti* [26]. Furthermore, the effects of irradiation on the gut-microbiome of *Ae. albopictus* were studied, revealing changes in diversity and density, with potential implications for the biological quality of sterile males released in SIT programs [27]. Both increases and decreases (in infection rates, survival, and sporozoite load) were also reported for the impact of irradiation on female *An. arabiensis*’ susceptibility to malaria parasites [28]. These findings highlight the importance of understanding the intricate effects of radiation on both male and female mosquitoes for the successful implementation of SIT strategies in mosquito vector control programs, and for assessing the risk of any female contamination in the males destined for release.

The sterilization process for mosquitoes in the SIT is currently being performed at either the pupal or adult stage [29–33]. Ideally, male pupae should be irradiated at a late stage, shortly before emergence as to not incur extensive off-target damage and maintain male biological quality. In the studies of Cunningham [24], Aldridge [22], and Moretti [20], irradiation was performed at the pupal stage only. In addition, longevity was assessed for females alone, although in the SIT, females would never be released alone but with a high number of males. We therefore aimed to assess the blood feeding rates of female mosquitoes of three species that have been irradiated at male-sterilizing doses at either the late pupal stage or adult stage, and that have been caged either with or without males to better understand the impact of high radiation doses and mating status on female blood feeding behavior and survival. The findings, together with the other few available reports, will help assess the risk associated with the accidental release of females that escape the sexing process during an SIT programme.

## Methods

### Mosquito strains

The *Ae. albopictus* strain used for the experiment originated from field collections in northern Italy and has been maintained at the Centro Agricoltura Ambiente, Bologna, Italy. The strain was transferred to the Insect Pest Control Laboratory (IPCL) of the FAO/IAEA Agriculture & Biotechnology Laboratories, Seibersdorf, Austria in 2010.

The *Ae. aegypti* strain originated from field collections in Juazeiro (Bahia), Brazil and was transferred to the IPCL from the insectary of Biofabrica Moscamed, Juazeiro, Brazil, in 2016. Both the *Aedes* strains have been maintained following the “Guidelines for Routine Colony Maintenance of *Aedes* mosquitoes” [34].

The Dongola strain of *An. arabiensis*, originating from Dongola, Northern State, Sudan, was donated by the Tropical Medical Research Institute, Khartoum, Sudan, in 2010 and has been maintained at the IPCL following the anopheline mass-rearing guidelines [35].

### Sample preparation and irradiation

Pupae of all three species were collected in 4 h windows to ensure late-stage, uniform age of 40–44 h for both *Aedes* species, and 20–24 h for *An. arabiensis*, i.e., the hours right before eclosion for both genera *Aedes* pupae were sexed on the basis of pupal size dimorphism using a glass-plate pupal sorter [3]. Pupae of *An. arabiensis* were sexed visually on the basis of terminalia using a stereomicroscope (Leica MZ16FA, Leica Microsystems GmbH, Wetzlar, Germany). For all species, batches of 20 female pupae and batches of 20 females + 20 male pupae were aliquoted into 20 mL plastic cups. Excess water was removed before irradiation at the doses required to sterilize males (over 99% induced sterility) for each species in known irradiation conditions, as has been established in previous studies: *Ae. albopictus* with 55 Gy [23], *Ae. aegypti* with 70 Gy [30], and *An. arabiensis* with 120 Gy [33]. These doses were selected according to data obtained from dose response curves established in the particular irradiator (Gammacell 220; Nordion Ltd, Kanata, Ontario, Canada), with a dose-rate of 52 Gy/min at the time of the experiments. After irradiation, all pupae batches were placed in 15 cm cubic Bugdorm^®^ cages (MegaView Science Co. Ltd., Taichung 40762, Taiwan) for emergence. Batches of 20 female pupae and 20 females + 20 male pupae that were not irradiated were used as controls.

Same batches of pupae as described above were placed in 15 cm cubic cages for emergence to obtain adults, which were then irradiated the following day in plastic tubes closed with a sponge with the same doses and irradiator as was used for pupae. The adults were then returned to their cages. All adults were supplied with a 10% sucrose solution.

For all species and treatments (non-irradiated controls, irradiated as pupae, irradiated as adults, with or without males), two true repetitions with each three technical repetitions were performed. The experiments were performed separately for each species and true repetition (i.e., not simultaneously). For the “mated” treatments, positive mating status is assumed. At a 1:1 male:female ratio in colony cages, former studies have shown high insemination rates. *Aedes aegypti* typically achieve very high insemination rates of 90–100%, [36, 37] as is observed in *Aedes albopictus* [38, 39]. Insemination rates have been found to be more variable in *Anopheles arabiensis* (70–90%, [40, 41]), however, in previous studies using this strain, insemination rates were closer to 90% [42].

### Dosimetry

The dosimetry system used to verify the dose received by the batches was based on Gafchromic HD-V2 and MD-V3 film (Ashland Advanced Materials, Bridgewater NJ, USA) following the protocol of the IAEA [43]. Three films of either HD film (for doses > 100 Gy) or MD film (for doses < 100 Gy) were packed in aluminum envelopes to keep them dry (Aluminum Laminate Detector Pouch FWT- 81, Far West Technologies, 330 S. Kellogg Ave Ste D, Goleta, CA 93117) and were placed directly above and below the pupae samples. Films were read with an optical density reader (DoseReader 4, RadGen, H-1118 Budapest, Sasadi út 36, Hungary) after 24 h of development.

### Blood feeding and engorgement rates

Preliminary test: females in all treatment cages (females alone, or females with males) were offered bloodmeals daily from day 1 without prior deprivation of the sugar source (starvation). The number of engorged females and total females was recorded. Following very poor response of the females of *Aedes* spp. with ages 1–3 days (none to very low feeding rates) and lack of usable data, the protocol was improved and is described in the next section. *Anopheles* females were attracted to the blood from day 1 and were motivated to feed daily, as has been shown in a previous study [4].

Improved protocol: for *Aedes* spp., blood feeding began in the afternoon when all *Aedes* spp. adults were aged 3–4 days old and continued every other day until they were 3 weeks old. The sugar solution was removed in the morning to enhance blood feeding activity. Both *Aedes* species were offered fresh porcine blood in collagen sausage casings, as is used in routine colony maintenance [34]. The *Anopheles* were offered thawed bovine blood daily from day 1, for 11 days, and then every other day for another week. The blood sausages were warmed in a warm water bath and placed on all cages of one technical repetition simultaneously and left for 20 min, after which the number of engorged females was recorded. The same was repeated for the second and third technical repetitions. The order of the cages bloodfed was alternated daily. Any dead females were removed from the cages and mortality was recorded.

### Longevity

A separate cohort of adults from all species were batched in groups of 20 females, or along with 20 males, and were either irradiated as adults as described above, or not irradiated (controls) and were kept in 15 cm cubic cages for the longevity study. Dead individuals were counted and removed on weekdays. In *An. arabiensis*, longevity was monitored until all the mosquitoes were dead; while in *Ae. albopictus* and *Ae. aegypti*, longevity was monitored until 54 days after irradiation, as they had a relatively high survival (up to 109 days in our preliminary experiments). Three repetitions were performed for each treatment group.

### Statistical analysis

All analyses were conducted in R (v. 4.3.1). The effect of irradiation treatment and mating status on blood feeding success (i.e., feeding rate) was analyzed using a binomial generalized linear mixed model built on Template Model Builder (*glmmTMB* function, *glmmTMB* package in R). Irradiation treatment (three levels: non-irradiated mosquitoes, irradiated pupae, and irradiated adults), mating status (two levels: unmated mosquitoes and mated mosquitoes), and their interactions were considered as fixed effects, and the replicate was considered as random effect.

Independent Samples *t*-test was used to compare overall engorgement rates in week 1 versus week 2. The *P* value was two-sided, and alpha levels were *P* < 0.05.

The effect of irradiation treatment and mating status on mosquito longevity was analyzed using a mixed effects Cox model (*coxme* function, *coxme* package in R). Irradiation treatment (two levels: non-irradiated mosquitoes and irradiated mosquitoes), mating status (two levels: unmated mosquitoes and mated mosquitoes), and their interactions were considered as fixed effects, and the replicate was considered as a random effect.

For model selection, we used the stepwise removal of terms, followed by likelihood ratio tests. Term removals that significantly reduced explanatory power (*P* < 0.05) were retained in the minimal adequate model. When needed, we performed post hoc pairwise comparisons with a *P*-value adjustment using Tukey method (*emmeans* function, *emmeans* package in R), to see differences between treatment groups.

As the dose of radiation used was different according to the species (55 Gy, 70 Gy, and 120 Gy used with *Ae. albopictus*, *Ae. aegypti* and *An. arabiensis*, respectively), the statistical analyses were performed for each species separately.

## Results

### Blood feeding rate

**Fig. 1:**
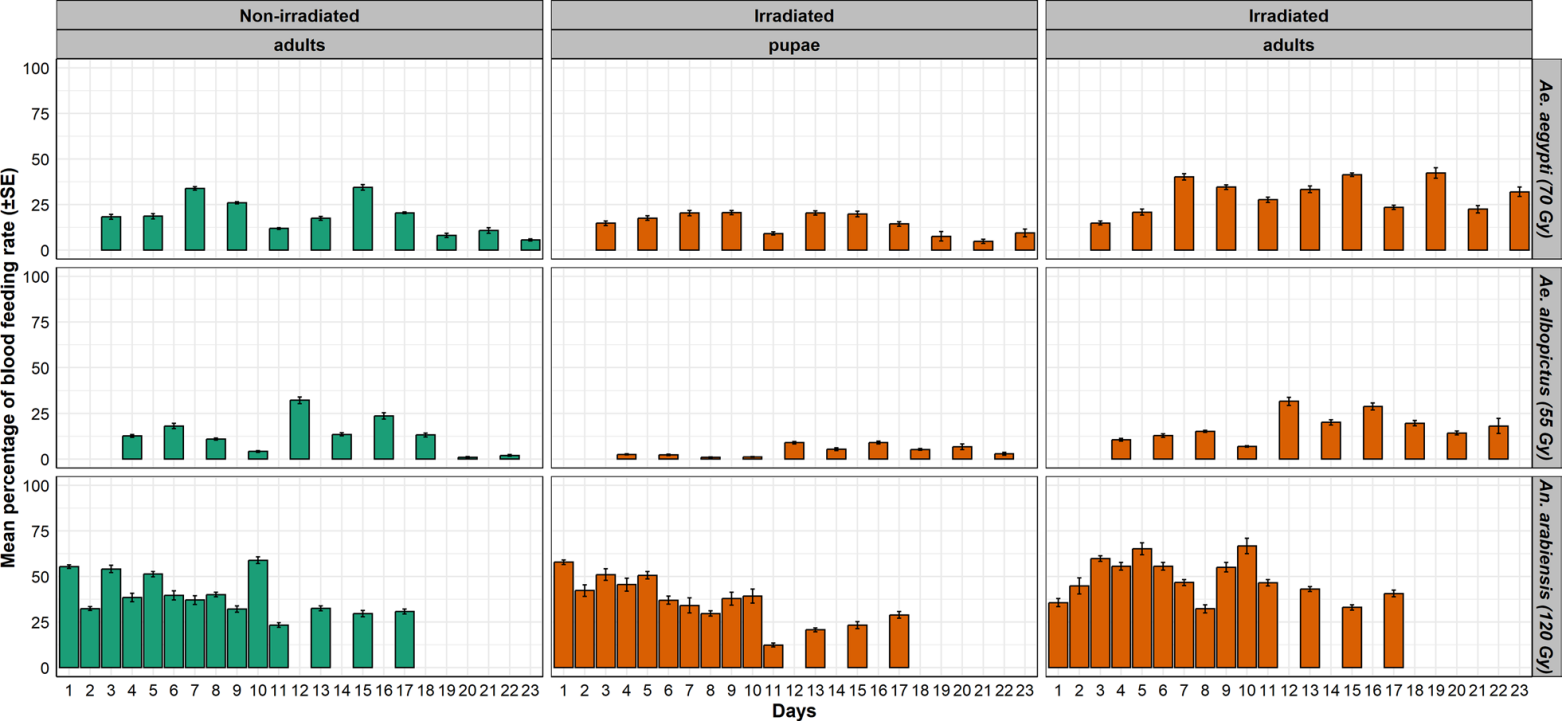
Daily engorgement rates for *Aedes aegypti, Aedes albopictus*, and *Anopheles arabiensis* irradiated as pupae or adults compared with non-irradiated controls

Generally, *Ae. albopictus* blood feeding activity was the lowest, and especially low for the first days and only started to increase in the second week. For all species, the activity showed a cyclic pattern, where peak engorgement rates were followed by a few days resting period, likely for those females that fed and were digesting the bloodmeal. All daily blood feeding rates for all species and treatments are shown in Fig. 1.

**Fig. 2:**
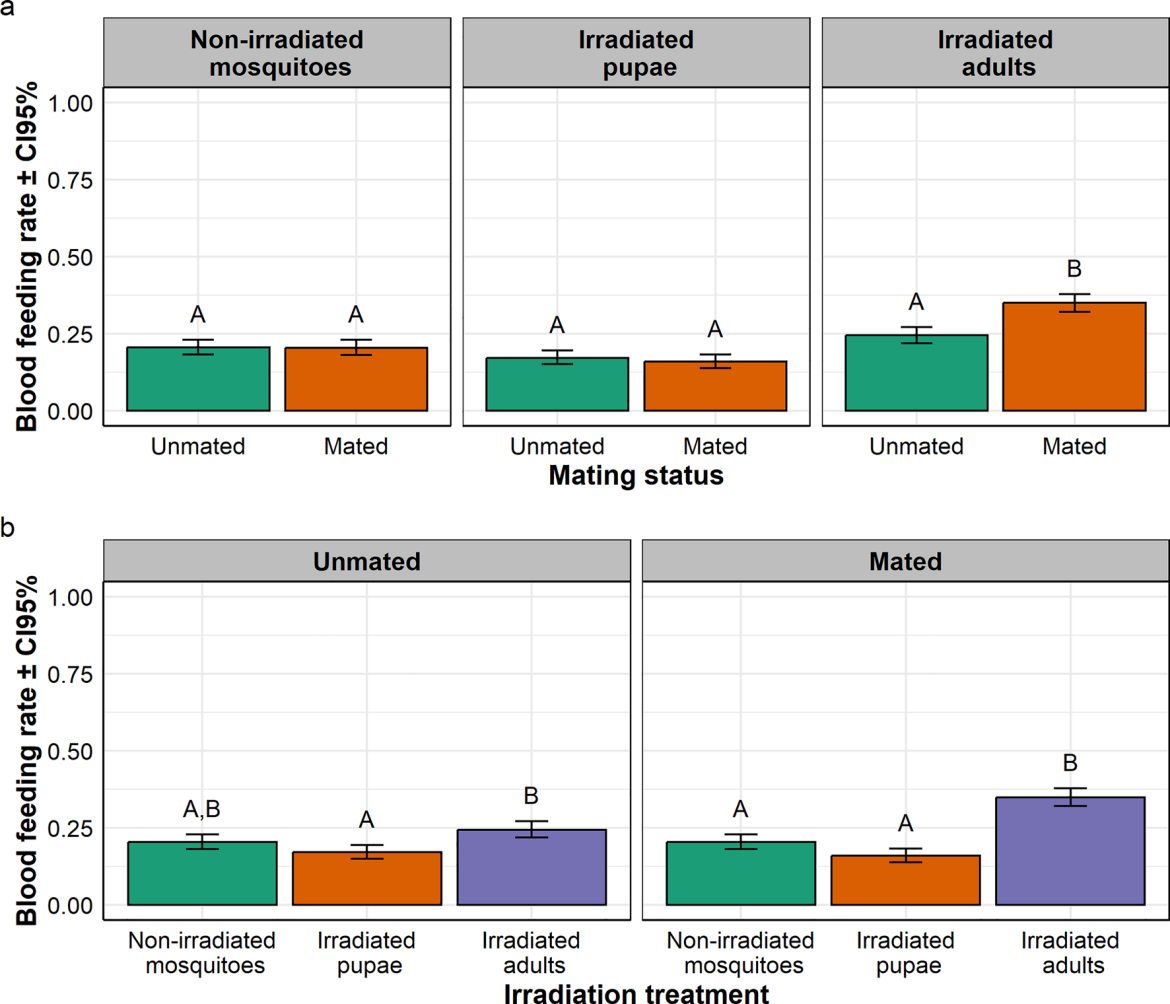
Effect of irradiation and mating status on **a** blood feeding rate in *Aedes aegypti*, and **b** interaction between irradiation treatment and mating status presented per irradiation treatment, and mating status, respectively. In each treatment group, bars with different letters are statistically different (*P* < 0.05)

In *Ae. aegypti,* the overall average daily feeding rates ranged from 15.91% to 34.92% according to the treatment. There was a significant interaction between irradiation treatment and mating status on the blood feeding rate (${x}_{2}^{2}$= 23.08, *P* < 0.001; Fig. 2a, b). In both the non-irradiated mosquito and irradiated pupa batches, feeding rate in mated mosquitoes was similar to that of unmated mosquitoes; while in the irradiated adult batch, feeding rate in mated mosquitoes was significantly higher than that of virgin mosquitoes (Fig. 2a). Considering the data from a different perspective, in the unmated mosquito batch, the feeding rate in irradiated adults was similar to that of non-irradiated mosquitoes, but significantly higher than that of irradiated pupae; while in the mated mosquito batch, feeding rates in irradiated adults were significantly higher than those in both non-irradiated mosquitoes and of irradiated pupae (Fig. 2b).

**Fig. 3:**
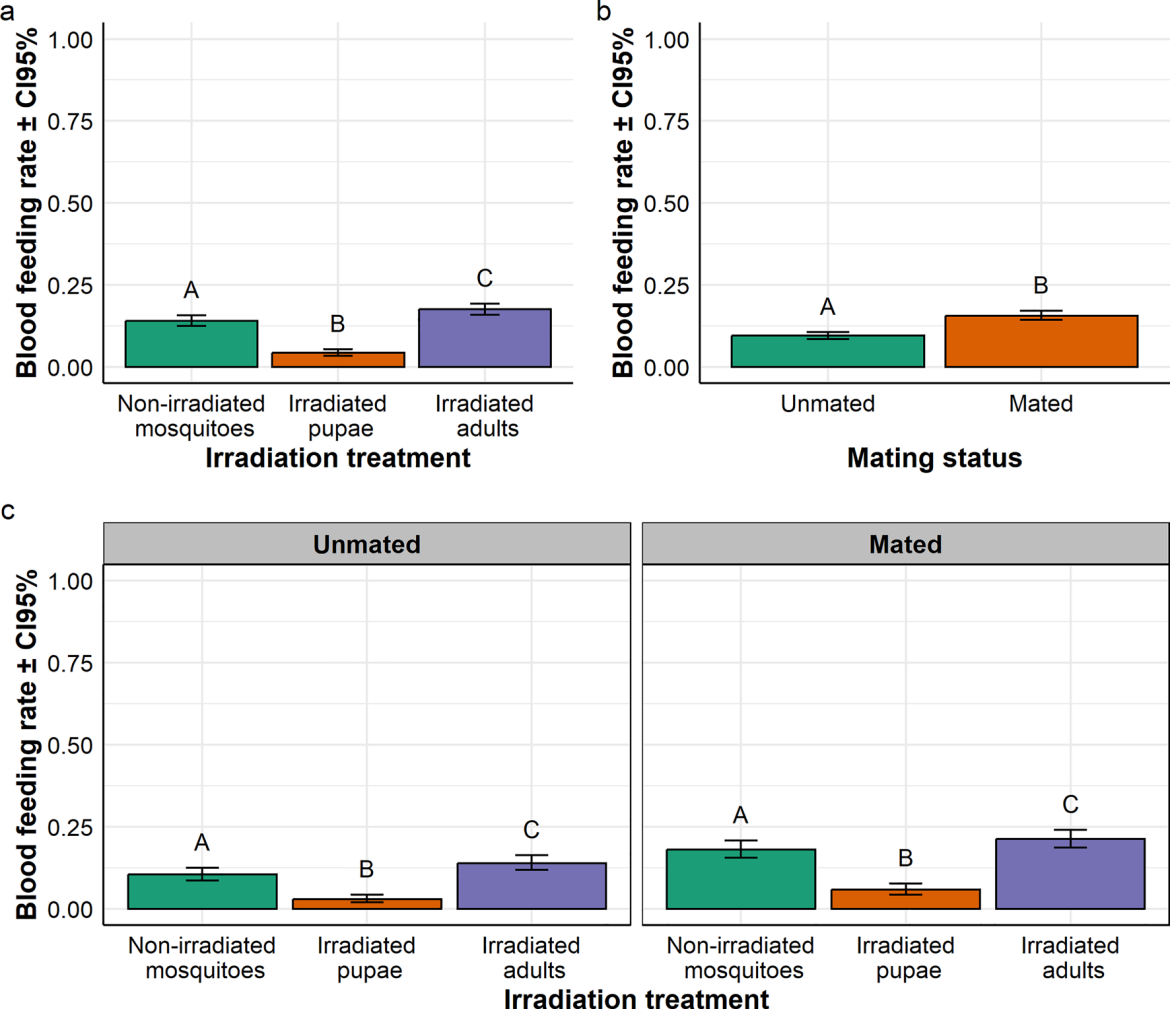
Effect of irradiation treatment and mating status on blood feeding rate in *Aedes albopictus.* Effect of **a** irradiation treatment and **b** mating status on the blood feeding rate. **c** Interaction between irradiation treatment and mating status on blood feeding presented per mating status. In each treatment group, bars with different letters are statistically different (*P* < 0.05)

In *Ae. albopictus,* the overall daily feeding rates ranged from 2.92% to 21.27% according to the treatment. There was a significant effect of the irradiation treatment on blood feeding rate (${x}_{2}^{2}$= 121.59, *P* < 0.001; Fig. 3a); the blood feeding rate in irradiated adults was significantly higher than those in both non-irradiated adults and in irradiated pupae, and the feeding rates in non-irradiated adults was significantly higher than that in irradiated pupae (Fig. 3a). There was a significant effect of the mating status on blood feeding rate (${x}_{1}^{2}$= 48.04, *P* < 0.001; Fig. 3b); the blood feeding rate in mated females was significantly higher than that in unmated ones (Fig. 3b). There was no interaction between irradiation treatment and mating status on blood feeding rate (${x}_{2}^{2}$= 0.82, *P* = 0.663; Fig. 3c); i.e., the feeding rate in irradiated adults was significantly higher than those in both non-irradiated mosquitoes and irradiated pupae, regardless of the mating status; and the feeding rates in mated mosquitoes was significantly higher than that in unmated ones, regardless of the irradiation treatment (Fig. 3C).

**Fig. 4:**
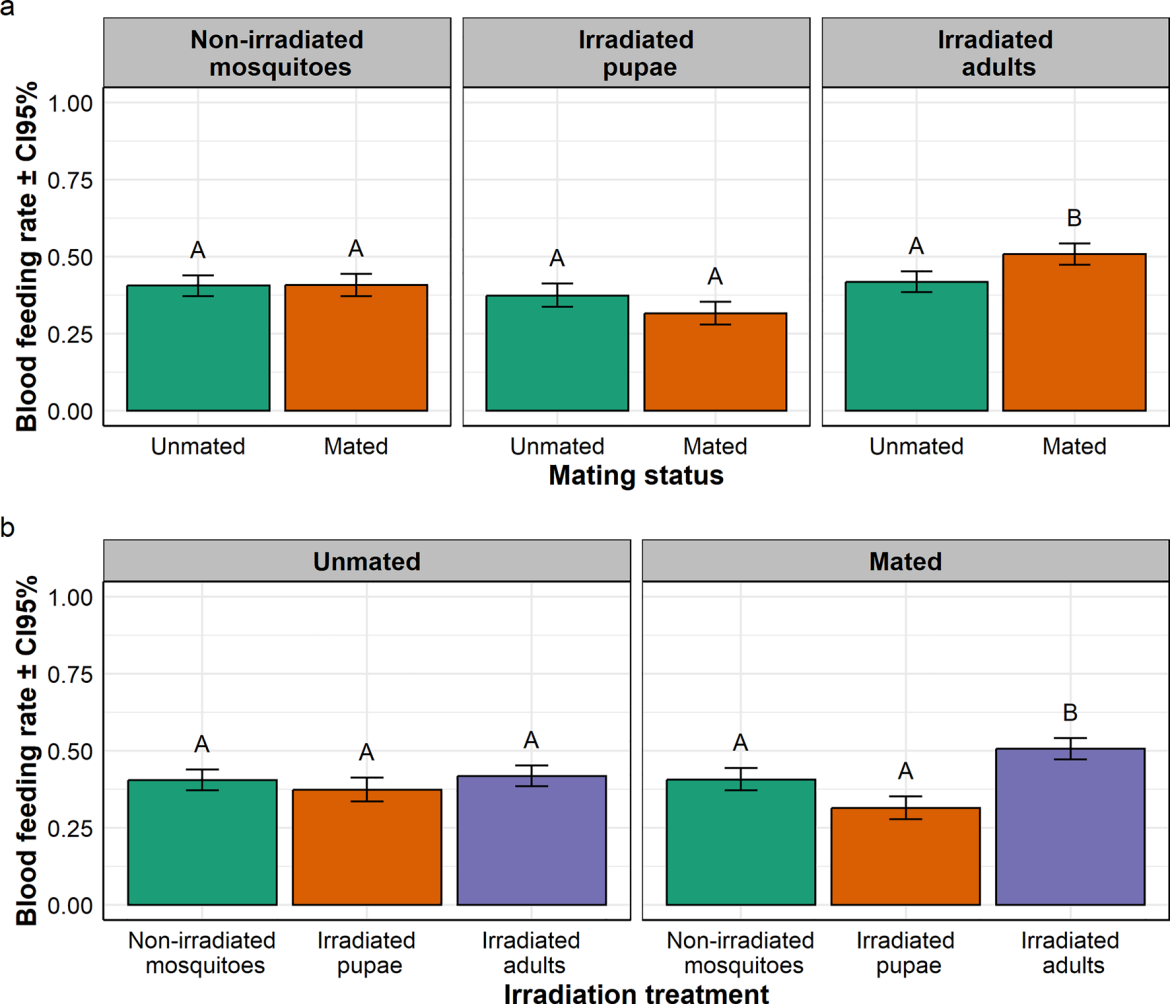
Effect of irradiation treatment and mating status on **a** blood feeding rate in *Anopheles arabiensis*, and **b** interaction between irradiation treatment and mating status on blood feeding rate presented per irradiation treatment and mating status, respectively. In each treatment group, bars with different letters are statistically different (*P* < 0.05)

In *Anopheles arabiensis,* the overall daily feeding rates ranged from 31.46% to 50.76% according to the treatment. There was a significant interaction between irradiation treatment and mating status on blood feeding rate (${x}_{2}^{2}$= 16.48, *P* < 0.001; Fig. 4a, b). In both the non-irradiated mosquito and irradiated pupa batches, the feeding rate in mated mosquitoes was not significantly different than that of unmated mosquitoes; while in the irradiated adult batch, feeding rate in mated mosquitoes was significantly higher than that in unmated mosquitoes (Fig. 4a). Expressed in another way, in the unmated mosquito groups, the blood feeding rate in irradiated adults was not statistically different from both those in non-irradiated mosquitoes and of irradiated pupae; while in the mated mosquito batch, feed rate in irradiated adults was significantly higher than both those in non-irradiated mosquitoes and in irradiated pupae (Fig. 4b).

### Comparison of engorgement rates in week 1 versus week 2

Following the report of Moretti et al. [20] in which biting rates were observed over 2 weeks and were found to be higher in week 2, we also looked at the overall blood feeding behavior in week 1 versus week 2 for each treatment group. For this, only the groups with mated females were analyzed (as accidentally released females are likely to be mated at blood feeding age).

It was observed that for *Ae. aegypti*, there was no significant difference in overall blood feeding rates in week 1 versus week 2 in the unirradiated controls, irradiated adults, and not significant for irradiated pupae (*df* = 28, *t* = −0.074, *P* = 0.940; *df* = 28, *t* = −11.174, *P* = 0.249; *df* = 28, *t* = −1.989, *P* = 0.056).

In week 1 of blood feeding, there was no significant difference between blood feeding rates of unirradiated controls and irradiated adults (*df* = 28, *t* = −1.291, *P* = 0.207). However, irradiated pupae fed significantly less than irradiated adults (*df* = 28, *t* = −3.181, *P* = 0.003) and unirradiated controls (*df* = 28, *t* = 2.836, *P* = 0.008).

In week 2, irradiated adults fed more frequently than controls (*df* = 28, *t* = −2.879, *P* = 0.007) and more than irradiated pupae (*df* = 28, *t* = 4.477, *P* < 0.001). There was no difference between irradiated pupae and controls (*df* = 28, *t* = 1.352, *P* = 0.186).

In *Ae. albopictus*, the behavior between the treatment groups was very similar to that of *Ae. aegypti*: there was no significant difference between blood feeding rates in week 1 versus week 2 for the unirradiated controls and the irradiated adults (*df* = 28, *t* = −0.700, *P* = 0.489; *df* = 28, *t* = −0.421, *P* = 0.676), but the irradiated pupae had slightly higher engorgement rates in week 2 than in week 1 (*df* = 28, *t* = −2.679, *P* = 0.012).

In week 1, unirradiated controls and irradiated adults showed the same engorgement rates (*df* = 28, *t* = −0.961, *P* = 0.344). Irradiated pupae blood fed significantly less frequently than the irradiated adults (*df* = 28, *t* = 2.39, *P* = 0.0230) and also less than the controls (*df* = 28, *t* = 2.278, *P* = 0.030).

In week 2, irradiated adults showed higher engorgement rates than the controls and the irradiated pupae (*df* = 28, *t* = −2.936, *P* = 0.007; *df* = 28, *t* = 3.785, *P* < 0.001). Irradiated pupae showed no difference to the controls (*df* = 28, *t* = 0.572, *P* = 0.571).

In *An. arabiensis*, fewer differences were observed between the treatment groups over the 2 weeks compared with the two *Aedes* species: unirradiated controls and irradiated adults showed no differences in engorgement rates in week 1 versus week 2 (*df* = 28, *t* = 1.668, *P* = 0.106; *df* = 28, *t* = 0.159, *P* = 0.874), whereas irradiated pupae showed a marked decrease in blood feeding activity in week 2 compared with week 1 (*df* = 28, *t* = 2.744, *P* = 0.010).

In week 1, irradiated adults and irradiated pupae showed indistinguishable engorgement rates (*df* = 28, *t* = 1.680, *P* = 0.103). Irradiated adults and irradiated pupae also did not significantly differ from controls (*df* = 28, *t* = −1.170, *P* = 0.251; *df* = 28, *t* = 0.945, *P* = 0.352).

In week 2, unirradiated controls and had similar blood feeding rates as irradiated adults and irradiated pupae (*df* = 28, *t* = −1.983, *P* = 0.57; *df* = 28, *t* = 1.159, *P* = 0.255), however, irradiated pupae showed a marked decrease in engorgement rates compared with irradiated adults (*df* = 28, *t* = 3.432, *P* = 0.001). A table summarizing the above results is provided in Supplementary file 1.

### Longevity of irradiated versus control adult females (mated and unmated)

**Fig. 5:**
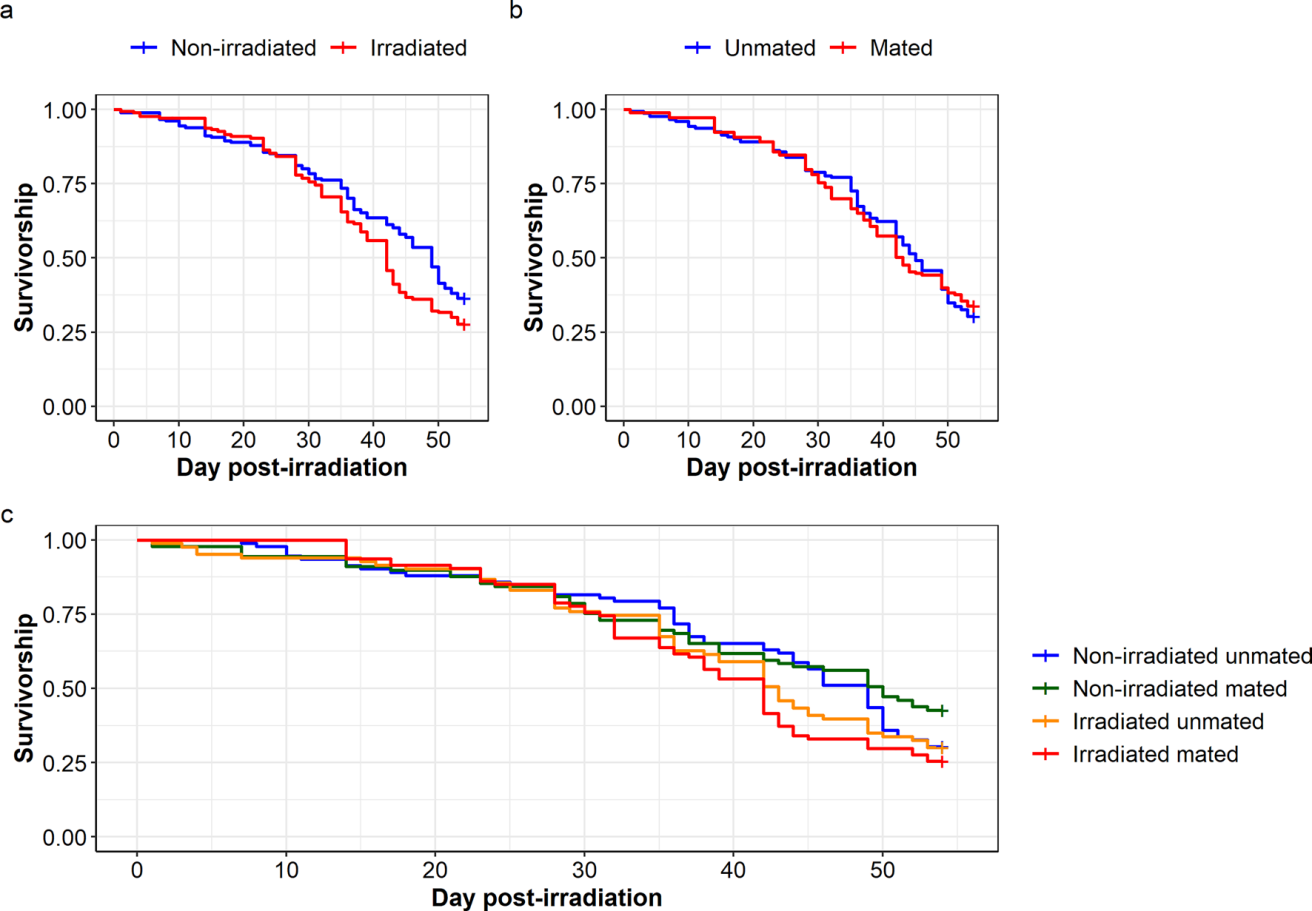
Effect of irradiation treatment and mating status on female *Aedes aegypti* survivorship. Effect of **a** irradiation treatment and **b** mating status on mosquito survivorship. **c** Interaction between irradiation treatment and mating status on mosquito survivorship. Survival monitoring was stopped on day 54 after irradiation. The “+” at the end of each curve indicates the censored data points

In *Ae. aegypti*, irradiation negatively influenced mosquito survivorship (${x}_{1}^{2}$= 4.93, *P* = 0.026; Fig. 5a). However, there was no significant effect of the mating status on mosquito survivorship (${x}_{1}^{2}$= 0.06, *P* = 0.809, Fig. 5b). There was no interaction between irradiation treatment and mating status on mosquito survivorship (${x}_{1}^{2}$= 1.88, *P* = 0.170; Fig. 5c). On day 21 and day 50 after irradiation, mosquito survival percentage ranged from 87.6% to 90.4% and from 29.8% to 47.2%, respectively, according to the treatment (Table 1).

**Table 1:** *Aedes aegypti* survival percentage on day 21 and day 50 after irradiation, according to the treatment

	Time (days)	*Aedes aegypti*							
		Unmated				Mated			
		Number at risk	Number of events	Survival percentage	[Lower–upper] 95% CI	Number at risk	Number of events	Survival percentage	[Lower–upper] 95% CI
Non-irradiated	21	81	11	88.0	[81.7–94.9]	80	11	87.6	[81.1–94.8]
	50	40	48	35.9	[27.3–47.1]	45	36	47.2	[37.9–58.8]
Irradiated	21	75	8	90.4	[84.2–96.9]	86	9	90.4	[84.7–96.6]
	50	29	47	33.7	[25.0–45.6]	28	57	29.8	[21.8–40.6]

**Fig. 6:**
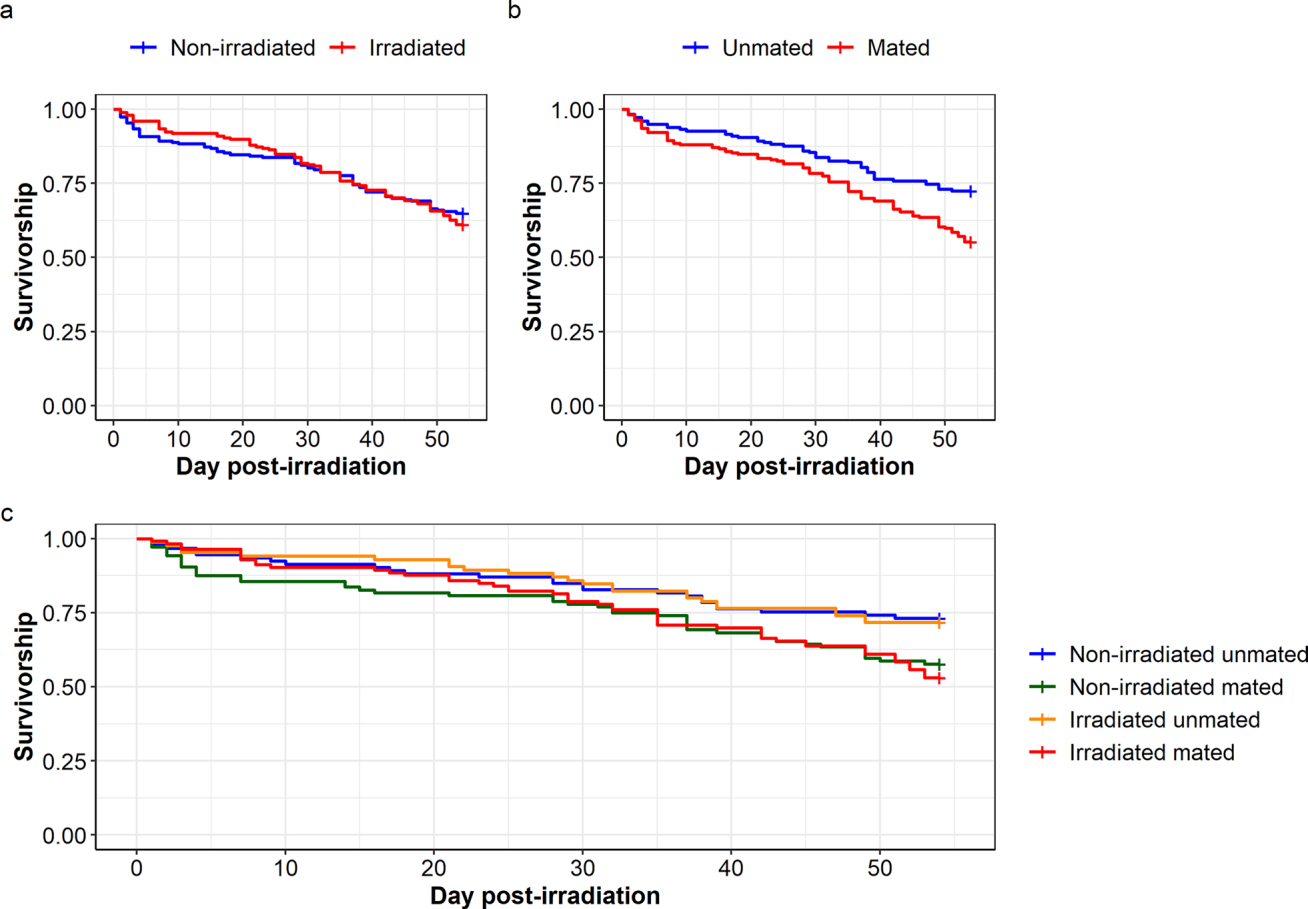
Effect of irradiation treatment and mating status on female *Aedes albopictus* survivorship. Effect of **a** irradiation treatment and **b** mating status on mosquito survivorship. **c** Interaction between irradiation treatment and mating status on mosquito survivorship. Survival monitoring was stopped on day 54 after irradiation. The “+” at the end of each curve indicates the censored data points

In *Ae. albopictus,* irradiation did not affect mosquito survivorship (${x}_{1}^{2}$= 0.13, *P* = 0.709; Fig. 6a). However, unmated mosquitoes lived longer than mated ones (${x}_{1}^{2}$= 10.96, *P* < 0.001; Fig. 6b). There was no interaction between irradiation treatment and mating status on mosquito survivorship (${x}_{1}^{2}$= 0.01, *P* = 0.1933, Fig. 6c). On day 21 and day 50 after irradiation, mosquito survival percentage ranged from 80.8% to 90.6% and from 58.7% to 74.2%, respectively, according to the treatment (Table 2).

**Table 2:** *Aedes albopictus* survival percentage on day 21 and day 50 after irradiation, according to the treatment

	Time (days)	*Aedes albopictus*							
		Unmated				Mated			
		Number at risk	Number of events	Survival percentage	[Lower–upper] 95% CI	Number at risk	Number of events	Survival percentage	[Lower–upper] 95% CI
Non-irradiated	21	82	11	88.2	[81.8–95.0]	85	20	80.8	[73.5–88.7]
	50	69	13	74.2	[65.8–83.6]	62	23	58.7	[49.9–68.9]
Irradiated	21	79	8	90.6	[84.6–97.0]	99	16	85.8	[79.6–92.5]
	50	61	16	71.8	[62.8–82.0]	69	28	61.1	[52.7–70.7]

**Fig. 7:**
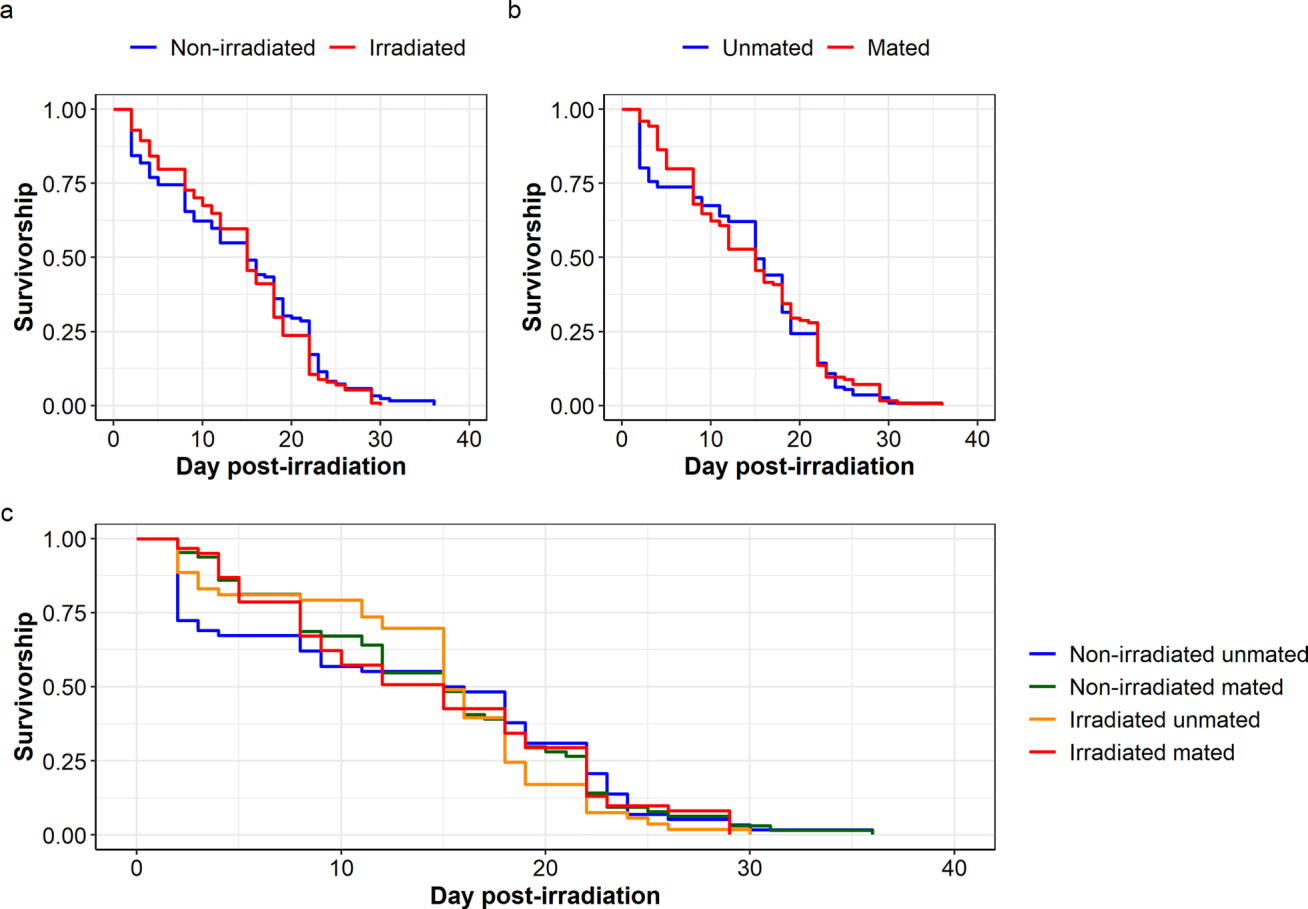
Effects of irradiation treatment and mating status on female *Anopheles arabiensis* survivorship. Effect of **a** irradiation treatment and **b** mating status on mosquito survivorship. **c** Interaction between irradiation treatment and mating status on mosquito survivorship

In *An. arabiensis,* neither irradiation nor mating status affected the mosquito survivorship (irradiation treatment: ${x}_{1}^{2}$=0.41, *P* = 0.519, Fig. 7a; mating status: ${x}_{1}^{2}$=0.08, *P* = 0.773, Fig. 7b). There was no interaction between irradiation treatment and mating status on mosquito survivorship (${x}_{1}^{2}$=0.01, *P* = 0.193, Fig. 7c). On day 14 and day 28 after irradiation, mosquito survival percentage ranged from 50.8% to 69.8% and from 1.8% to 8.2%, respectively, according to the treatment (Table 3).

**Table 3:** *Anopheles arabiensis* survival percentage on day 14 and day 28 after irradiation, according to the treatment

	Time (days)	*Anopheles arabiensis*							
		Unmated				Mated			
		Number at risk	Number of events	Survival percentage	[Lower–upper] 95% CI	Number at risk	Number of events	Survival percentage	[Lower–upper] 95% CI
Non-irradiated	14	32	26	55.1	[43.7–69.6]	35	29	54.6	[43.7–68.4]
	28	3	29	5.1	[1.7–15.6]	4	31	6.2	[2.4–16.1]
Irradiated	14	37	16	69.8	[58.4–83.3]	31	30	50.8	[39.7–65.0]
	28	1	36	1.8	[0.2–13.1]	5	26	8.2	[3.5–19.0]

### Longevity of irradiated females in the blood feeding rate experiment

As the total number of females per blood fed cage was recorded to establish the engorgement rates, the mortality data could be extracted and analyzed.

**Fig. 8:**
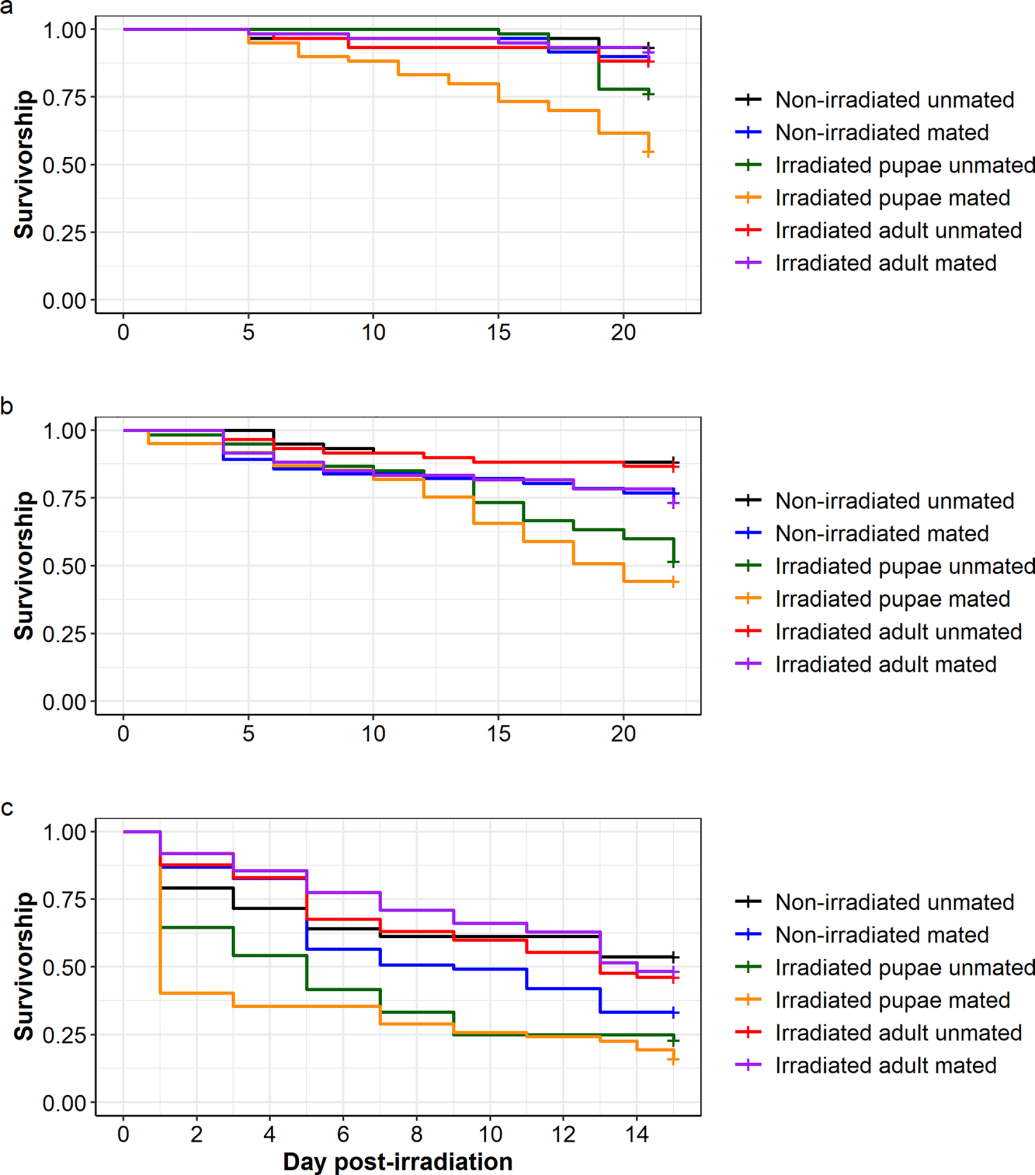
Effects of irradiation treatment and mating status on bloodfed female **a** *Aedes aegypti,* **b** *Aedes albopictus*, and **c** *Anopheles arabiensis* survivorship; “no” and “pres” indicates no males present or males present (i.e., unmated or mated) and “irr” indicates irradiation treatment

During the blood feeding rate experiment for *Ae. aegypti*, the survival of females irradiated as adults did not differ from the non-irradiated control group, regardless of the presence of males (*P* > 0.05). The presence of males also did not significantly affect survival of the control females, nor the irradiated adulttreatment groups. However, females irradiated as pupae (unmated) showed slightly reduced survival compared with the unmated controls (*P* = 0.020), and mated females irradiated as pupae had a significantly higher risk of dying than the unmated control females (exp(coef): 8.652; *P* < 0.001) (Fig. 8a).

In *Ae. albopictus*, the presence of males had a more visible impact on female longevity in the irradiated treatment groups. Females irradiated as adults that were caged with males (mated) showed slightly reduced survival (*P* = 0.043), whereas the risk of death for females irradiated as pupae (unmated and mated) was 5–6 times higher than unirradiated, unmated control females (exp(coef): 5.296; *P* < 0.001) and (exp(coef): 6.563; *P* < 0.001), (Fig. 8b).

In *An. arabiensis* females, the survival of females irradiated as adults (with and without males) was similar to unirradiated, unmated control females (*P* = 0.057). Females irradiated as pupae without males (unmated) had twice the risk of dying compared with the controls (exp(coef): 2.374; *P* < 0.001) but survived slightly better than the mated group, which were more than three times at risk of dying (exp(coef): 3.144; *P* < 0.001), (Fig. 8c).

## Discussion

The ability to blood feed and to survive long enough to incubate and transmit pathogens are two of the key factors that determine a mosquito’s vectorial capacity and need to be investigated following various treatments the females are exposed to, in the event that they are accidentally released into the wild. As the irradiation process can be done both at the pupal and adult stages, and mating status can affect blood feeding behavior [44], this study compared blood feeding rates of mated versus virgin female mosquitoes that have been irradiated at male-sterilizing doses at either pupal or adult stage.

Our findings reveal distinct species-specific responses to irradiation and mating status in terms of blood feeding behavior and longevity. Overall, *Ae. albopictus* exhibited the lowest blood feeding activity, particularly in the first week, consistent with its slower behavioral activation post-emergence. Across all species, blood feeding followed a cyclical pattern, suggesting rest periods for bloodmeal digestion. In *Ae. aegypti*, blood feeding rates were significantly influenced by an interaction between irradiation treatment and mating status. Notably, mated females irradiated as adults displayed significantly higher feeding rates compared with their virgin counterparts and with females irradiated at the pupal stage. This suggests a possible synergistic effect of mating and adult-stage irradiation on host-seeking and feeding motivation. Similar patterns were observed in *An. arabiensis*, where mated, irradiated adults also showed elevated feeding rates, highlighting that adult-stage irradiation does not universally suppress feeding behavior and may even slightly enhance it in mated females. In contrast, *Ae. albopictus* feeding was independently influenced by both irradiation and mating, without a significant interaction. Irradiated adults consistently showed higher feeding rates, while mated females fed more than unmated ones across all treatments. This uniformity suggests that *Ae. albopictus* blood feeding is less sensitive to the interaction of these two variables, though still susceptible to each independently.

*Aedes* females irradiated as pupae showed a marked decrease in feeding rates compared with the other treatment group and controls in week 1. There was no significant difference between controls and irradiated adult feeding behavior in the three species in the first week, but the frequency of blood feeding generally increased compared with controls in week 2. Although no data are currently available on the survival of released females in the field, we speculate that similar reductions in longevity are expected compared with released males, which are reported to not survive beyond a week, with mean survival of sterile males reported to be between 2 and 5 days for the three species [45–48]. This pattern was less pronounced in *An. arabiensis*, where irradiated adults did not feed more in week 2, but irradiated pupae fed less in the second week, unlike for the *Aedes* spp., where feeding increased in week 2 in this treatment group. It is important to note that the overall female feeding rate (proportion of females engorged/total possible blood feeding events) in this study lay between 40% and 50% during the duration of the experiment. Thus, it cannot be ascertained whether and when the females took two consecutive blood meals. This aspect is important, as these females can become vectors only if they are capable of having at least two consecutive blood meals surviving at least 7–14 days between the two feedings [26]. In other words, if blood feeding occurs on day 1 and again on day 2, this would be irrelevant for disease transmission, whereas blood feeding on days 1 and 10 would be relevant.

Longevity analyses showed that for *Ae. aegypti* females, irradiation reduced survivorship, which was expected, as females are significantly more radiosensitive than their male counterparts [22, 28, 49], but mating had no effect, aligning with some previous findings that sterilization can impair survival independently of reproductive behavior. An interaction of mating and irradiation may be important, as it has been reported that mating can increase the survivorship in this species [50]. In *Ae. albopictus*, unmated females lived significantly longer than mated ones, and unlike *Ae. aegypti*, irradiation had no discernible impact on longevity. For *An. arabiensis*, neither irradiation nor mating status significantly affected survivorship, although survival dropped dramatically by day 28 across all treatments.

During the blood feeding experiments, survival trends were generally similar to those observed in non-blood-fed groups. However, females irradiated as pupae, particularly those that were mated, consistently showed reduced survival across all species. In *Ae. albopictus* and *An. arabiensis*, mated females irradiated at the pupal stage faced the highest mortality risks—up to sixfold increases compared with controls—suggesting that both irradiation timing and post-emergence reproductive stress play critical roles in shaping longevity outcomes.

Our results were only partially consistent with the three main studies focusing on this topic [20, 22, 24], in which irradiation has been shown to impact the blood-feeding behavior of female mosquitoes in complex and sometimes contradictory ways, with engorgement rates or biting attempts increasing or decreasing in the different studies, with effects also depending on species, developmental stage at the time of irradiation, radiation dose, experimental design, blood source, and physiological factors such as mating status.

In the studies by Aldridge et al. [22] and Cunningham [24], gamma irradiation at the pupal stage significantly reduced survivorship, blood-feeding behavior, and oviposition in *Aedes aegypti* females. The studies found that irradiated females showed a decreased propensity to take a blood meal, lowered overall feeding success, and delayed initiation of feeding compared with non-irradiated controls. These effects were dose-dependent and consistent with the hypothesis that irradiation damages somatic tissues, including the nervous system and salivary glands, potentially impairing the sensory and motor functions required for host location and blood ingestion. In both reports, the results demonstrated a dose-dependent decrease in blood-feeding activity, and at a sterilizing dose of 50 Gy, only approximately 10% of the irradiated females successfully took a blood meal, compared with higher feeding rates in non-irradiated controls.

These findings are consistent with our results, suggesting that gamma irradiation of pupae at doses sufficient to sterilize male mosquitoes also significantly impairs the blood-feeding behavior of females. This reduction in feeding propensity may mitigate the potential risk associated with the inadvertent release of irradiated females in SIT programs, as their likelihood to seek hosts and transmit pathogens is substantially diminished.

Contrastingly, Moretti et al. [20] reported an increase in the number of bites per female across repeated feeding opportunities in both Ae. aegypti and Ae. albopictus. Differences between studies may arise from variation in irradiation source (X-ray versus gamma), dose, mosquito strain, and experimental design. Our study, which used gamma irradiation at male-sterilizing doses, did not observe a comparable increase in blood feeding rates.

Vector competence in mosquitoes is determined by multiple barriers that a pathogen must overcome: infection of the midgut, dissemination through the hemocoel, replication in the salivary glands, and eventual transmission during blood feeding. These processes are shaped by both survival and feeding behavior, as well as immune and physiological responses. Recent studies have shown that irradiation can influence these parameters in complex and sometimes opposing ways. For example, da Silva et al. [51] reported that gamma irradiation reduced *Ae. aegypti* competence for Zika virus, while Trefry et al. [52] found increased susceptibility to Mayaro virus in irradiated *Ae. aegypti*. Zhang et al. [53] further demonstrated dose-dependent effects in *Ae. albopictus*, with moderate doses enhancing arbovirus infection but higher sterilizing doses reducing it. Taken together, these studies suggest that although irradiation can modify infection dynamics, females exposed to high sterilizing doses—as would be the case in SIT programs—are generally less likely to pose a meaningful risk of pathogen transmission compared with untreated wild females. This is because their survival is substantially reduced, feeding success is impaired, and competence is not consistently enhanced at operational dose levels.

However, it is important to acknowledge that there remains substantial uncertainty regarding how female contamination rates translate into epidemiological risk. Our study was not designed to evaluate this. While operational SIT programmes often use contamination values such as 1% as practical guidelines, these should be interpreted as operational rather than biologically or epidemiologically derived thresholds. In this manuscript, we restrict our conclusions to the biological traits measured. At the same time, SIT programmes continue to emphasize the importance of minimizing accidental female release through ongoing improvements in sex-separation technologies, operational procedures, and complementary mitigation strategies aimed at reducing any residual disease transmission risk. These mitigation approaches include treating released cohorts via blood meals spiked with endectocides such as ivermectin [4, 54], the use of antiviral or antiparasitic compounds as was done for sterile tsetse [55, 56], or modification of vector competence via symbiotic bacteria [57], and the integration of incompatible insect technique (IIT) with SIT to suppress or block pathogen transmission in any remaining females [39, 58, 59]. Continued development of efficient, robust, and scalable genetic sexing strains further remains a central priority [60, 61].

A more recent study by Zhang et al. [62] on mating harassment suggests that high ratios of sterile males to females can also suppress mosquito populations through behavioral interference. Under laboratory conditions, it was observed that when the male-to-female ratio exceeded 50:1, female mosquitoes experienced increased mortality and reduced blood-feeding success. This was attributed to persistent mating attempts by males in cages and shortly after release, which disrupted females’ ability to feed and led to energy depletion. In semi-field and field trials, similar effects were noted. Notably, in a 1.17-hectare field trial in China, the female biting rate decreased by 80%, and female mosquito density dropped by 40%. These reductions were linked to male swarming behavior around humans, which impeded females from feeding effectively. The study concludes that mating harassment by sterile males not only induces sterility, but also directly reduces female mosquito survival and biting rates. This impact could reduce the risk caused by accidentally released females, since they will be exposed to high male-to-female ratios and thus have a low survival.

## Conclusions

In the context of SIT, the accidental release of a small number of irradiated females is considered a manageable but nonnegligible risk regarding disease transmission. Irradiation generally reduces female mosquito survival, fecundity, and in many cases blood-feeding success [22, 24], which together lower their overall vectorial capacity compared with wild females.

Rigorous sex separation methods and quality control are essential to minimize female release rates, which emphasizes the need to prioritize the development of genetic sexing strains or any other tool that can eliminate the release of females or reduce the risk of pathogen transmission during field applications.

## Supplementary Information

Supplementary material 1.

## Data Availability

Data supporting the main conclusions of this study are included in the manuscript.
