# Supplementary material for: Impact of male-sterilizing doses and mating status on blood feeding rates and longevity in Aedes aegypti, Aedes albopictus, and Anopheles arabiensis females
Source: Parasit Vectors. 2026 Mar 21;19:208. doi: 10.1186/s13071-025-07242-8 (PMC13169929; doi:10.1186/s13071-025-07242-8)
Supplement: Supplementary file 1 — Supplementary material 1. [file 13071_2025_7242_MOESM1_ESM.docx]

# Supplementary file: Summary of Engorgement Rate Comparisons

## *Aedes aegypti*

### Week-to-week

| Treatment | Week 1 vs Week 2 | p-value | Interpretation |
| --- | --- | --- | --- |
| Control | No difference | 0.940 | Stable across weeks |
| Irradiated adults | No difference | 0.249 | Stable |
| Irradiated pupae | Not significant (marginal) | 0.056 | Slight, non-significant increase |

### Week 1

| Comparison | Result | p-value |
| --- | --- | --- |
| Control vs irradiated adults | No difference | 0.207 |
| Irradiated pupae vs irradiated adults | Pupae fed less | 0.003 |
| Irradiated pupae vs controls | Pupae fed less | 0.008 |

### Week 2

| Comparison | Result | p-value |
| --- | --- | --- |
| Irradiated adults vs controls | Adults fed more | 0.007 |
| Irradiated adults vs irradiated pupae | Adults fed more | <0.001 |
| Irradiated pupae vs controls | No difference | 0.186 |

## *Aedes albopictus*

### Week-to-week

| Treatment | Week 1 vs Week 2 | p-value | Interpretation |
| --- | --- | --- | --- |
| Control | No difference | 0.489 | Stable |
| Irradiated adults | No difference | 0.676 | Stable |
| Irradiated pupae | Higher in Week 2 | 0.012 | Increased feeding |

### Week 1

| Comparison | Result | p-value |
| --- | --- | --- |
| Control vs irradiated adults | No difference | 0.344 |
| Irradiated pupae vs irradiated adults | Pupae fed less | 0.023 |
| Irradiated pupae vs controls | Pupae fed less | 0.030 |

### Week 2

| Comparison | Result | p-value |
| --- | --- | --- |
| Irradiated adults vs controls | Adults fed more | 0.007 |
| Irradiated adults vs irradiated pupae | Adults fed more | <0.001 |
| Irradiated pupae vs controls | No difference | 0.571 |

## *Anopheles arabiensis*

### Week-to-week

| Treatment | Week 1 vs Week 2 | p-value | Interpretation |
| --- | --- | --- | --- |
| Control | No difference | 0.106 | Stable |
| Irradiated adults | No difference | 0.874 | Stable |
| Irradiated pupae | Lower in Week 2 | 0.010 | Decreased feeding |

### Week 1

| Comparison | Result | p-value |
| --- | --- | --- |
| Irradiated adults vs irradiated pupae | No difference | 0.103 |
| Irradiated adults vs controls | No difference | 0.251 |
| Irradiated pupae vs controls | No difference | 0.352 |

### Week 2

| Comparison | Result | p-value |
| --- | --- | --- |
| Irradiated adults vs controls | No difference | 0.570 |
| Irradiated pupae vs controls | No difference | 0.255 |
| Irradiated pupae vs irradiated adults | Pupae fed less | 0.001 |
